# The effect of opioid-free anesthesia on the quality of recovery after gynecological laparoscopy: study protocol for a prospective randomized controlled trial

**DOI:** 10.1186/s13063-021-05166-z

**Published:** 2021-03-12

**Authors:** Jae Yen Song, Hoon Choi, Minsuk Chae, Jemin Ko, Young Eun Moon

**Affiliations:** 1 Department of Obstetrics and Gynecology, Seoul St. Mary’s Hospital, College of Medicine, The Catholic University of Korea, 222 Banpo-daero, Seocho-gu, Seoul, 137-701 Republic of Korea; 2 Department of Anesthesiology and Pain Medicine, Seoul St. Mary’s Hospital, College of Medicine, The Catholic University of Korea, 222 Banpo-daero, Seocho-gu, Seoul, 137-701 Republic of Korea

**Keywords:** Opioid-free anesthesia, Opioid-induced hyperalgesia, Opioid-related adverse effects, Quality of recovery, Gynecological laparoscopy

## Abstract

**Background:**

Because of the indiscriminate use of opioids during the perioperative period, opioid-free anesthesia (OFA) has been increasingly required. Nevertheless, the studies on the detailed techniques and effects of OFA are not sufficient. The Quality of Recovery-40 (QoR-40) questionnaire is a validated assessment tool for measuring recovery from general anesthesia. However, no study has used the QoR-40 to determine if OFA leads to better recovery than standard general anesthesia. Therefore, we aim to perform this study to determine the effects of OFA using dexmedetomidine and lidocaine on the quality of recovery as well as the various postoperative outcomes.

**Methods:**

The participants (*n* = 78) will be allocated to one of the two groups; the study group will receive bolus and infusion of dexmedetomidine and lidocaine, and the control group will receive remifentanil infusion during general anesthesia for gynecological laparoscopy. The other processes including anesthetic and postoperative care will be performed similarly in the two groups. Intraoperative hemodynamic, anesthetic, and nociceptive variables will be recorded. Postoperative outcomes such as QoR-40, pain severity, and opioid-related side effects will be assessed. Additionally, an ancillary cytokine study (inflammatory cytokine, stress hormone, and reactive oxygen species) will be performed during the study period.

**Discussion:**

This will be the first study to determine the effect of OFA, using the combination of dexmedetomidine and lidocaine, on the quality of recovery after gynecological laparoscopy compared with standard general anesthesia using remifentanil. The findings from this study will provide scientific and clinical evidence on the efficacy of OFA.

**Trial registration:**

ClinicalTrials.gov NCT04409964. Registered on 28 May 2020

## Background

Laparoscopy is a common surgical treatment for various gynecologic diseases. CO_2_ pneumoperitoneum is necessary for this surgery, which requires general anesthesia. Because of the pneumoperitoneum and surgical stimulus, the routine use of intravenous (IV) opioids during general anesthesia tends not to be questioned.

Increasingly indiscriminate use of opioids during the perioperative period ultimately led to an “opioid crisis,” particularly in the USA [1, 2]. Among patients receiving chronic opioid therapy, treatment is started after surgery in 27% of cases on the prescription by surgeons or anesthesiologists [3]. Therefore, they have been confronted with their responsibilities for considerate use as perioperative opioid prescribers [2].

For the recent 10 years, opioid-free postoperative analgesia is needed; many studies have reported on this topic over the past 10 years [4]. Multimodal analgesia using *N*-methyl-d-aspartate (NMDA) antagonists, local anesthetics, anti-inflammatory drugs, and alpha-2 agonists can be effective. However, studies on opioid-free anesthesia (OFA) are still in the early stages [5].

While OFA using dexmedetomidine has been reported to be effective during several types of surgery, such as bariatric surgery and laparoscopic cholecystectomy [6, 7], there has been no report on gynecological laparoscopy. Patients undergoing this surgery are generally sensitive to pain and are at high risk of postoperative nausea and vomiting (PONV). Moreover, tolerance to pain develops within 90 min with remifentanil, a commonly used opioid for general anesthesia [8], leading to the requirement for more opioids during the acute postoperative period. Thus, patients scheduled for gynecological laparoscopy are at risk of ever-increasing opioid requirements and opioid-related side effects, such as PONV, sedation, or ileus, leading to delayed recovery from surgery.

The Quality of Recovery-40 (QoR-40) questionnaire is used to assess recovery from general anesthesia according to five dimensions of health, including physical comfort, physical independence, emotional state, psychological support, and pain. Generally, a 10-point difference equates to a 15% improvement in the quality of recovery [9]. The validity and reliability of the QoR-40 have been confirmed in previous studies, and it has been used to investigate recovery after various anesthetic and surgical techniques [10, 11]. However, no study has used the QoR-40 to determine if OFA leads to better recovery than standard general anesthesia. Therefore, we aim to perform this study to determine the effects of OFA using dexmedetomidine and lidocaine on the quality of recovery from gynecological laparoscopy, according to various postoperative outcomes. Additionally, we will assess the feasibility of OFA for gynecological laparoscopy.

## Methods

**Fig. 1:**
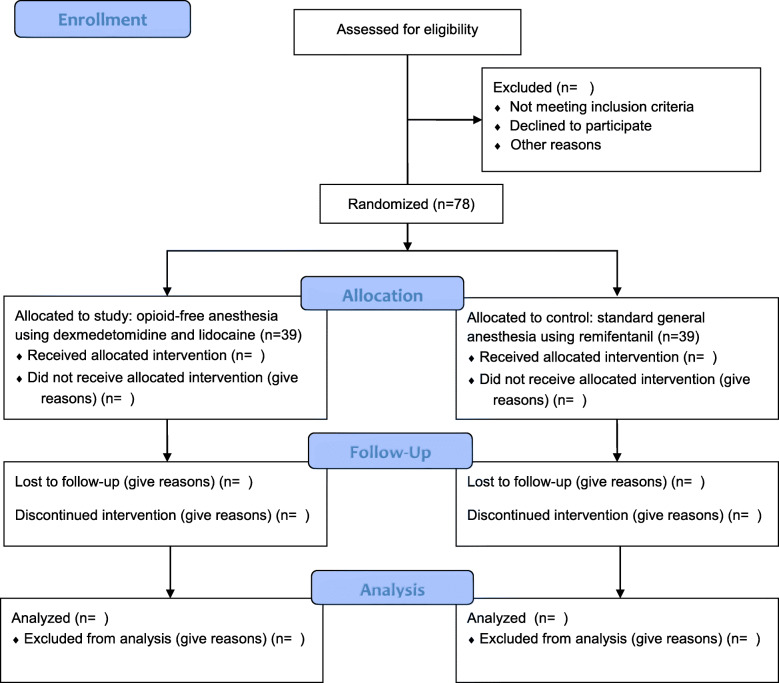
Flow diagram

**Fig. 2:**
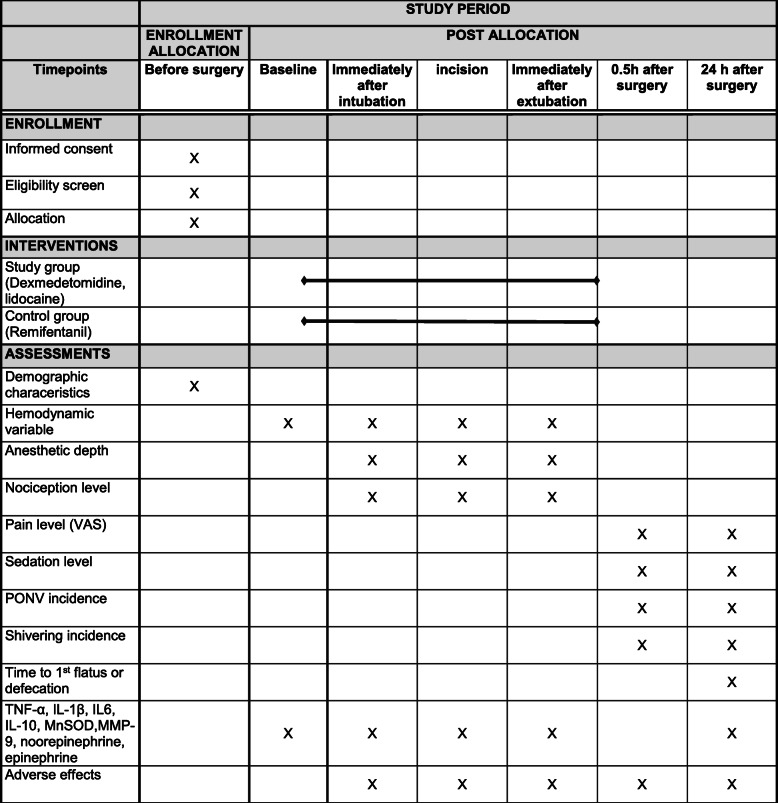
Standard Protocol Items: Recommendations for Interventional Trials (adapted from SPIRIT figure). VAS, visual analog scale; TNF, tumor necrosis factor; IL interleukin; MnSOD, manganese superoxide dismutase; MMP, matrix metalloproteinase

This randomized, single-blind clinical trial of patients scheduled to undergo elective gynecological laparoscopy will use concealed allocation. The patients will be allocated to receive either OFA (dexmedetomidine and lidocaine) or a standard anesthesia protocol (remifentanil). This study will be conducted at Seoul St. Mary’s Hospital, Catholic University Medical College, South Korea. Figures 1 and 2 provide an overview of the study schedule, which was designed in accordance with the Standard Protocol Items: Recommendations for Interventional Trials (SPIRIT) guidelines.

### Participants

The inclusion criteria for this study are as follows: adults (aged 20–65 years) scheduled for elective gynecological laparoscopy including hysterectomy, uterus myomectomy, oophorectomy, salpingectomy, cyst enucleation, and cystectomy. Postoperative IV patient-controlled analgesia (PCA) will be applied in all the patients. The exclusion criteria are refusal to participate in the study, emergent surgery, cancer surgery, chronic pain requiring a pain killer, psychiatric disease, preoperative bradycardia (heart rate [HR] < 50 bpm), hypotension, atrioventricular block, intraventricular or sinoatrial block, body mass index > 35 kg/m^2^, allergy or history of adverse events to study drug, pregnancy, or lactation.

### Randomization and blinding

The enrolled participants will be randomized to one of two groups (study or control group). The block randomization scheme will be generated using a web-based random number generator (at www.random.org) by a research nurse. Participants will be randomized to receive OFA or standard general anesthesia at a 1:1 allocation ratio using stratified block randomization with a fixed block size. Once a patient has been enrolled, the medical staff will open an opaque, sequentially numbered envelope containing the group allocation.

The participants and surgeons will be kept blinded to the group allocation throughout the study period. The medical staff providing postoperative care and evaluating outcomes in the post-anesthesia care unit (PACU) and the ward will also be unaware of the group allocation. The anesthesiologists performing general anesthesia will be the only unblinded staff. However, they will not participate in the postoperative care or assessment of postoperative outcomes.

### Intervention

This study has two arms (Fig. 1). Both arms include general anesthesia for gynecological laparoscopy and postoperative care, according to the clinical practice guidelines. Before starting the study, the participants will be instructed on how to assess their pain intensity using a visual analog scale (VAS; 0 cm = no pain, 10 cm = worst pain imaginable) and how to use IV PCA. To improve adherence to the protocol, participants will be asked to request analgesia without hesitation if the VAS pain score is > 4.

None of the patients will receive premedication. Electrocardiography, non-invasive blood pressure measurements, pulse oximetry, neuromuscular monitoring using train-of-four (TOF) stimulation, bispectral index (BIS VISTA Monitoring System; Aspect Medical Systems, Inc., Norwood, MA, USA) monitoring of anesthesia depth, and Surgical Pleth Index (SPI; General Electric Healthcare, Helsinki, Finland) measurements of nociception will be applied in the operating room. General anesthesia will be induced with 1.5–2 mg/kg IV propofol. After unconsciousness is confirmed (BIS value < 60), absence of an eyelash reflex, and no response to verbal stimulation, 0.8 mg/kg rocuronium will be injected and orotracheal intubation will be performed using a direct laryngoscope when there are zero TOF twitches. Ventilation will be controlled mechanically and then adjusted to maintain the end-tidal CO_2_ value at 25–40 mmHg throughout the surgery. Additional rocuronium will be administered as required. Anesthesia will be maintained with 4–6% desflurane (expired concentration) in 40% air/oxygen (total flow, 4 L/min) to maintain the BIS at 30–60. Ephedrine (4 mg IV) will be injected in the cases with systolic blood pressure (SBP) < 80 mmHg or mean arterial pressure (MAP) < 60 mmHg. If the HR decreases to < 45 bpm, atropine (0.25 mg IV) will be administered.

All the enrolled patients will be allocated to one of the following two groups: study group (OFA using dexmedetomidine and lidocaine) and control group (standard general anesthesia using remifentanil).

The study group will receive dexmedetomidine 0.7 μg/kg IV for 10 min before the propofol injection. Immediately after inducing anesthesia, a 1.5-mg/kg IV lidocaine bolus will be injected followed by a 1.5-mg/kg/h infusion. The dexmedetomidine infusion will be started at 0.5 μg/kg/h and adjusted in steps of 0.1 μg/kg/h to maintain the SBP baseline within ±20%. The dexmedetomidine and lidocaine infusion will be stopped when skin suturing begins.

The control group will receive 3.5 ng/ml remifentanil using a target-controlled infusion (Orchestra Base Primea, Fresenius Vial, Brezins, France) before the propofol injection. After inducing anesthesia, the remifentanil infusion will be adjusted in increments of 0.5 ng/ml to maintain the SBP baseline within ±20%. The remifentanil infusion will be stopped at the end of skin suturing.

The laparoscopy will be performed under video guidance with three punctures in the abdomen. Intraperitoneal pressure will be maintained at about 12 mmHg. All patients will receive 5 mg dexamethasone at the start, and palonosetron 75 μg at the end, of surgery to prevent PONV. Acetaminophen (1 g via an IV drip) and ketorolac (30 mg IV) will be administered 30 min before the end of surgery for postoperative pain control. These non-opioid analgesics will be also used in the general ward. After confirming self-respiration, patients will be extubated and transferred to the PACU. If the patient complains of pain (VAS score > 4) in the PACU, fentanyl 0.5–1 μg/kg will be administered immediately. Once the acute pain is under control, IV PCA (fentanyl 15 μg/kg in 100 mL normal saline, basal rate 0 mL/h, bolus 1 mL, lock-out time 10 min) will be applied in all patients, and no loading dose will be administered. Metoclopramide 10 mg IV will be injected in cases of PONV. The patients will be discharged to the general ward when their Aldrete score is ≥9 [12].

### Outcomes

#### Primary outcome

The primary outcome is the quality of postoperative recovery according to the QoR-40 questionnaire scores on postoperative day (POD) 1. The QoR-40 questionnaire includes five dimensions of recovery: physical comfort (12 items), emotional state (9 items), physical independence (5 items), psychological support (7 items), and pain (7 items). Each item is scored on a 5-point Likert scale (none of the time, some of the time, usually, most of the time, and all of the time). The total score on the QoR-40 ranges from 40 (poorest possible recovery) to 200 (best possible recovery). The QoR-40 will be completed the day before the surgery and on POD 1.

#### Other outcomes

To determine whether OFA provides the same effects as remifentanil-used general anesthesia, such as hemodynamic stability and sedation, we will obtain intraoperative hemodynamic data (SBP, MAP, and HR), anesthetic depth (BIS), and nociception severity (SPI) data, at baseline, before intubation (at the time of unconsciousness), immediately after intubation, at the time of the incision, and immediately after tracheal extubation. Additionally, to assess intraoperative complications, episodes of bradycardia (< 45 bpm) in association with the administration of atropine, hypotension (SBP < 80 mmHg or MAP < 60 mmHg), hypertension (MAP > 90 mmHg), and shock (anaphylactic, septic, cardiac, or hemorrhagic) will be recorded.

Pain severity will be assessed using a VAS upon arrival in the PACU and every 15 min thereafter. Additionally, the sedation severity (none/sedated and responsive to verbal stimuli/sedated and unresponsive to verbal stimuli), the incidence of PONV and shivering, the requirement for analgesics and antiemetics, and the PACU stay will be assessed. These outcomes will be evaluated again 24 h after the surgery. Additionally, the time to the first flatus or defecation will be recorded.

### Ancillary cytokine study

To determine the potential involvement of OFA in neuroinflammation, an ancillary study will be performed on the 50 enrolled patients (*n* = 25 in each group). Blood samples will be measured for cytokines at baseline, immediately after tracheal intubation, at the time of the incision, immediately after tracheal extubation, and 24 h after surgery. The cytokines to be measured include pro-inflammatory cytokines (tumor necrosis factor-α, interleukin [IL]-1β, and IL-6), an anti-inflammatory cytokine (IL-10), reactive oxygen species (ROS; manganese superoxide dismutase, matrix metalloproteinase [MMP]-9), and stress hormones (norepinephrine and epinephrine). All blood samples will be placed in tubes and centrifuged within 1 h, and the plasma will be separated and stored at − 70 °C until analysis. All plasma specimens will be discarded after the completion of the study.

### Sample size

The primary outcome is the QoR-40 score on POD 1. Based on a previous study that reported a 13-point difference in QoR-40 scores between different anesthetic techniques [13], a sample size of 34 was calculated to be as necessary to achieve a power of 80% with a type 1 error of 0.05. An additional 15% of participants are added to account for possible loss to follow-up. Thus, the final sample size will be 78 participants (39 in each group).

### Statistical analysis

A researcher blinded to the group allocation will perform the statistical analysis of all randomized patients (intention-to-treat analysis) using the SPSS for Windows software (ver. 18.0; SPSS Inc., Chicago, IL, USA). Data will be expressed as frequencies or percentages for categorical variables and means with standard deviations for quantitative variables. The Kolmogorov-Smirnov test will be used to check the normality of the distribution of the quantitative variables. Student’s *t* test or the Mann-Whitney *U* test will be used to analyze the quantitative variables, including the primary outcome (QoR-40) and postoperative fentanyl requirement. The chi-square or Fisher’s exact test will be used to analyze the qualitative variables. Continuous endpoints repeatedly measured during the study period will be analyzed using repeated measures two-way analysis of variance. A *P* value < 0.05 will be considered significant.

Analyses will be performed between the groups as randomized. Participants who withdraw from the trial will be followed up, according to the routine clinical practices. To reduce missing data in the intention-to-treat analysis, the investigator may ask the participants which specific aspects of the trial they want to withdraw from.

Missing data will be tested if they are missing at random; otherwise, the last observation carried forward method will be applied [14]. A sensitivity analysis will also be performed to check for inconsistencies. A subgroup analysis will be performed to check for differences in the outcomes according to the surgeon. No interim analyses are planned, and no serious adverse effects are expected to arise during the study, because the protocols have been used previously without any such effects [6, 7, 15].

### Data collection and monitoring

Clinical data will be entered into paper-based case report forms. After each assessment, the identifiers (e.g., name and birth date) will be anonymized, coded, and stored on a secure server. The files will be backed up on a password-protected computer. Data will be handled according to Korean law.

The project team designed and prepared the trial and will disseminate the results. The team will meet every month to discuss the progress of the study. A data monitoring committee, comprising two independent professors (anesthesiologists) and a physician pharmacologist, will meet three times a year throughout the study. This committee is responsible for safeguarding the interests of the trial participants, assessing the safety of the interventions, and monitoring the overall conduct of the trial. Any deviation from the protocol will be documented in a report. All significant protocol modifications will be communicated to the relevant parties and updated in the trial register.

### Dissemination plan

The results obtained from this study will be disseminated at anesthesia conferences (local and international meetings). The key findings will be reported in the trial registry. A full study report will be submitted for publication in an anesthesia journal, preferably an open-access journal.

## Discussion

This will be the first study to determine the effect of OFA, using the combination of dexmedetomidine and lidocaine, on the quality of recovery after gynecological laparoscopy compared with standard general anesthesia using remifentanil. The findings from this study will provide scientific and clinical evidence on the efficacy of OFA.

Remifentanil is used for general anesthesia due to its rapid metabolism and washout. However, this unique pharmacologic characteristic is linked with the development of opioid-induced hyperalgesia (OIH). A meta-analysis including 27 studies reported significant increases in acute pain after general anesthesia with remifentanil, leading to higher morphine requirements [16]. The roles of ROS and inflammation in OIH have attracted much attention [17]. It has been reported that remifentanil stimulates the overproduction of pro-inflammatory cytokines and ROS, leading to the activation of neuronal NMDA receptors, which play an important role in OIH [18, 19]. Also, excessive MMP-9 activity induced by remifentanil mediates extracellular matrix abnormalities, which can lead to a variety of neuropathological conditions including neuroinflammation and hyperalgesia [17, 20–22]. However, these effects have only been reported in animal studies.

Dexmedetomidine, a potent α2 agonist, is a unique drug with sedative, analgesic, anti-shivering, and anesthetic-sparing effects. In addition, this drug has been reported to reduce inflammation and stress [23]. Although the underlying mechanism is not well understood, several possible mechanisms have been suggested, including attenuation of cytokine production and inhibition of apoptosis and central sympatholytic effects [24, 25]. It is highly plausible that this drug not only reduces surgical stress in a similar manner to remifentanil, but also alleviates neuroinflammation (unlike remifentanil). Therefore, this drug could serve as a cornerstone of OFA.

Lidocaine has been widely used in clinical practice due to its sympatholytic and analgesic effects. Moreover, it is reported to decrease inflammatory cytokine levels [26]. Therefore, lidocaine also has positive effects on OFA. Generally, low-dose infusion (1–2 mg/kg/h for < 6 h) of lidocaine does not cause adverse effects [15].

Intraoperatively, general anesthesia should provide hemodynamic stability and deep sedation, which are generally ensured via conventional monitoring. However, intraoperative monitoring, i.e., nociceptive monitoring, has not been used routinely. We will measure the SPI for nociceptive monitoring, which has not been attempted in any other OFA study. The SPI ranges between 0 (low sympathetic tone) and 100 (high sympathetic tone). The SPI in the range of 20–50 is regarded to reflect an appropriate level of nociception [27]. This index has been widely used for several years and is reportedly more valid than other nociceptive measures, such as the pupillary pain index and nociception level (NoL) [28].

In addition, this study aims to determine whether OFA during the postoperative period has positive effects on clinical outcomes such as acute postoperative pain, shivering, and PONV, where such effects might lead to better recovery after surgery. These clinical outcomes will be supported by the measurement of pro-inflammatory cytokines and ROS levels.

In summary, this study will assess the feasibility and effects of OFA, using dexmedetomidine and lidocaine, during gynecological laparoscopy. Given the increasing requirement for OFA, along with a deficiency in scientific and clinical evidence of its efficacy, this study will provide useful information on this analgesic modality.

## Trial status

This study was approved by the Institutional Review Board of Seoul St. Mary’s Hospital Ethics Committee (KC20MNSI0130) on 7 April 2020 and registered at ClinicalTrials.gov (NCT04409964) on 28 May 2020. The recruitment of participants started in June 2020. The anticipated recruitment period is 12 months. This protocol is version 2.0 in June 2020.

## Data Availability

The data generated in this study can be shared after a reasonable request to the corresponding author.

## Abbreviations

BIS: Bispectral index
HR: Heart rate
IL: Interleukin
IV: Intravenous
MAP: Mean arterial pressure
MMP: Matrix metalloproteinase
NMDA: *N*-methyl-d-aspartate
OFA: Opioid-free anesthesia
PACU: Post-anesthesia care unit
PCA: Patient-controlled analgesia
PONV: Postoperative nausea and vomiting
QoR-40: Quality of Recovery-40
ROS: Reactive oxygen species
SBP: Systolic blood pressure
SPI: Surgical Pleth Index
TOF: Train-of-four
VAS: Visual analog scale
